# Negative human papillomavirus testing in normal smears selects a population at low risk for developing high-grade cervical lesions

**DOI:** 10.1038/sj.bjc.6601726

**Published:** 2004-04-20

**Authors:** C Clavel, J Cucherousset, M Lorenzato, S Caudroy, J M Nou, P Nazeyrollas, M Polette, J-P Bory, R Gabriel, C Quereux, P Birembaut

**Affiliations:** 1 Laboratoire Pol Bouin, Hôpital Maison Blanche, CHU de Reims, 45 rue Cognacq-Jay, Reims 51100, France; 2 Department of Statistics, CHU de Reims, Reims 51100, France; 3 Department of Obstetrics and Gynaecology, CHU de Reims, Reims 51100, France

**Keywords:** HPV, cervical cancer, screening

## Abstract

High-risk human papillomaviruses (HR-HPV) are the necessary cause of cervical carcinomas and there is an increasing interest in using HR-HPV DNA detection in adjunction to cytological examination for primary cervical screening. To determine whether women with a normal smear negative for HR-HPV DNA detection with the Hybrid Capture II assay might represent a low-risk population for developing a high-grade squamous intraepithelial lesion (HSIL), 4401 women have been followed in a period of 12–72 months (median=34 months). During this follow-up, four HSIL and one microinvasive carcinoma have been detected in this cohort (three in the cohort of 3526 women >29 years). The global negative predictive value (NPV) of double-negative tests is thus of 99.9% (ninety-five percent confidence interval (95% CI): 99.8–100%), whereas cytology alone gives an NPV of 99.2% (95% CI: 98.9–99.5%). If we obtain a second negative HR-HPV test 1–2 years after the initial test, the NPV is 100%. The NPV is also of 100% in the cohort of women >49 years. We conclude that all these women could be safely screened at longer intervals between 3 and 5 years. This policy will offset the increased costs induced by an additional HR-HPV testing in primary screening.

It is now well established that oncogenic (high-risk) human papillomaviruses (HPV) are a necessary causal factor in the development of cervical intraepithelial and invasive neoplasias (Lorincz *et al*, 1992; Bosch *et al*, 1995; Walboomers *et al*, 1999; Zur Hausen, 2002). Infections with high-risk HPV (HR-HPV) are associated with a relative risk of between 8 and 11 for the development of squamous intraepithelial lesions (SIL) (Gaarenstroom *et al*, 1994). Moreover, only low-grade SIL (LSIL) containing HR-HPV progress to high-grade SIL (HSIL) (Koutsky *et al*, 1992). Owing to this, there is an increasing interest in using HPV DNA detection either alone or in addition to classic cytological examination for primary cervical screening (Cuzick *et al*, 1995, 1999, 2003; Meijer *et al*, 1998; Clavel *et al*, 1999, 2001; Kuhn *et al*, 2000; Ratnam *et al*, 2000; Schiffman *et al*, 2000; Schneider *et al* 2000; Kjaer *et al*, 2002; Petry *et al*, 2003; Lorincz and Richart, 2003; Sherman *et al*, 2003). Most authors consider that a positive HPV testing selects a population at high risk for developing an HSIL, while a negative HPV testing has a very good negative predictive value (NPV). Indeed, considering that the mean time from detectable LSIL to preclinical invasive cancer is 12–13 years (Gustafson and Adami, 1989), Meijer *et al* (1998) have proposed that women with cytologically normal smears and a negative HR-HPV test could be rescreened every 8 years.

Hybrid Capture-II (HC-II), a commercial HPV detection test, (Digene, Gaithersburg, MD, USA) was introduced 7 years ago (Lorincz, 1996). Hybrid Capture-II is a nonradioactive, reproducible, relatively rapid, liquid hybridisation assay in microtiters designed to detect 18 HPV types divided into high-risk (types 16, 18, 31, 33, 35, 39, 45, 51, 52, 56, 58, 59 and 68) and low-risk (types 6, 11, 42, 43 and 44) groups. The sensitivity of this assay is quite similar to that of polymerase chain reaction (PCR) (Peyton *et al*, 1998). Recently, the Food and Drug Administration in the USA has authorised the use of this assay in women aged 30 years and older for primary screening in adjunction to cytology. In a recent preliminary study, using this test in women with normal smears, we have shown that recurrent infection detected with HC-II was associated with a relative risk of incident HSIL of 237.3 when HR-HPV test remained positive at two controls. In contrast, in the cohort of 2432 women testing negative for HR-HPV infection followed on a period of 60 months, only two women developed an HSIL (Bory *et al*, 2002). Thus the NPV of a negative test with HC-II could be useful in primary screening when associated with a smear within normal limits.

At the present time, there are few longitudinal studies with a detailed and prolonged follow-up of women with a normal smear with a negative HR-HPV testing (Kjaer *et al*, 2002; Sherman *et al*, 2003). Thus, the aim of the present study is to evaluate on a large population of women with an initial smear within normal limits the usefulness and limitations of a negative HR-HPV testing in such women. For that, we have followed on a period from 12 to 72 months 4401 women with a normal smear and a negative HR-HPV testing at enrolment. The end point in all these women was the detection of an HSIL at the histological examination.

## MATERIAL AND METHODS

### Study population

A total of 4401 women with a median age of 41 years (range 15–79 years) were recruited for the study between August 1997 and November 2002. This population was restricted to women who underwent their routine screening in the Department of Obstetrics and Gynaecology of the CHU of REIMS. We excluded subjects on the basis of a recent cytological abnormality and/or an untreated cervical lesion in the past 2 years and patients with AIDS. All women were informed of the aim of the study and gave their consent. The follow-up of these women was between 12 and 72 months (median=34 months).

### Cytologic diagnosis

At the first gynaecologic examination, in 1278 women, two samples were taken: first, a cytological smear with an Ayre's spatula, for classical cytology, then one scrape for the HC-II test with a Cervexbrush (Medscan, Uppsala, Sweden). These samples were suspended in 1 ml of specimen transport medium for HPV testing (Digene, Gaithersburg, MD, USA). In another group, 3123 women had only one cervical scrape with a Cervexbrush at the first entry. Samples were prepared for liquid-based cytology with the ThinPrep technique (Cytyc Corporation, Marlborough, MA, USA) and 4 ml of the sample were used for HPV testing. In total, considering the first samples and the follow-up of women, we collected 10 641 cervical smears and scrapes, including 9112 liquid-based samples. Smears were classified according to the Bethesda system for reporting cervical or vaginal cytological diagnosis. We selected women with adequate smears including metaplastic and/or endocervical cells according to the criteria of Bethesda, which represented 93% of our total smears. However, even if the smear was not considered as adequate, we also included women with smears with cytological abnormalities. The cytotechnicians and pathologists involved in the study were not informed about the results of the HPV testing. All smears showing cytological abnormalities and biopsy specimens were examined by the same two independent pathologists, without knowledge of cytology results for the biopsies examination. The results were compared and if the first two diagnoses disagreed, a third pathologist reviewed the case with no knowledge of preceding diagnoses. Consensus diagnoses were determined by two-thirds majority when possible and remaining discrepancies resolved by conference review. Patients with HSIL were systematically treated by loop electrosurgical excision procedure (LEEP). Data from these LEEP specimens were included in the disease definitions.

### Colposcopic referral

**Figure 1:**
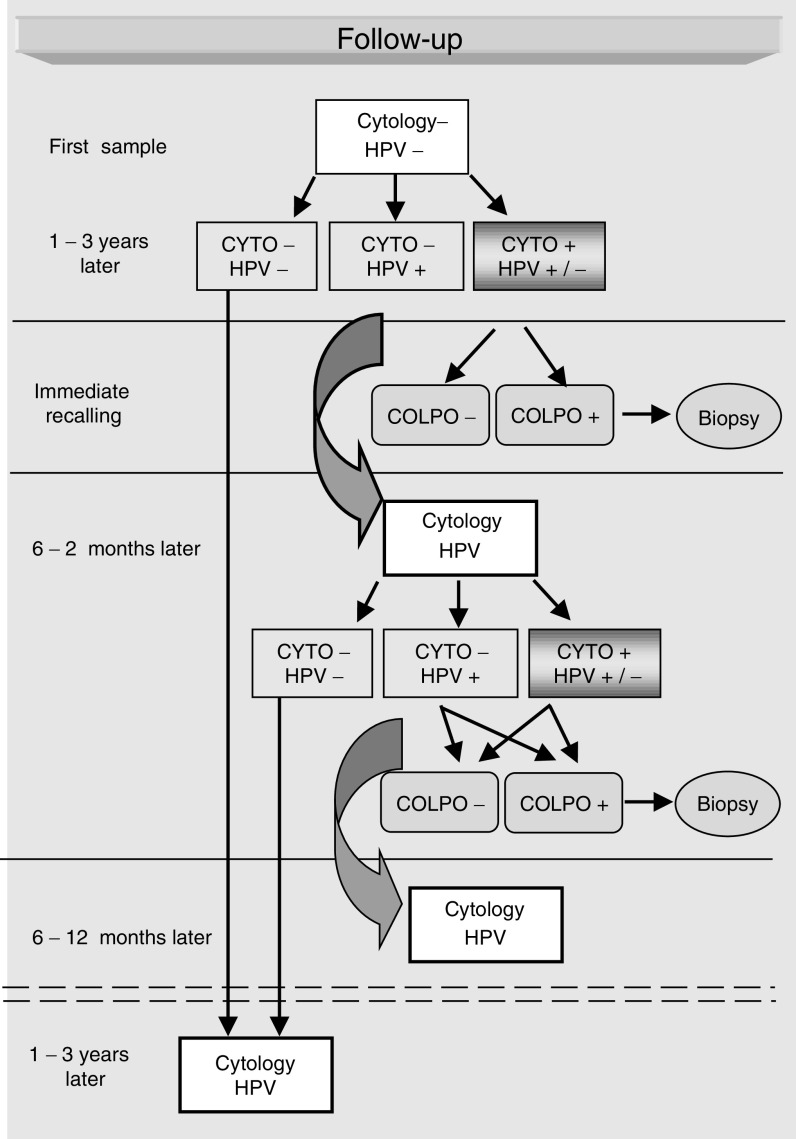
Protocol of follow-up of women with normal cytology and a negative HR-HPV testing.

In our protocol (Figure 1

), all the women presenting in their follow-up cytological abnormalities (from ASCUS to HSIL) were systematically recalled for colposcopy in the next few weeks, at an interval ranging from 14 to 160 days (mean 95 days) after entry examination. Punch biopsy specimens were taken from the areas colposcopically suspicious for cervical intraepithelial neoplasia (CIN). All the women with smears within normal limits but presenting an HR-HPV infection were also systematically recalled 6–12 months later for a new cytological examination and HR-HPV testing followed by colposcopy if a lesion and/or a persistent HR-HPV infection was detected. Punch biopsy specimens were taken from the areas colposcopically suspicious for CIN. The women with a second HR-HPV test positive without any detectable lesions were recalled 6–12 months later for a third control with cytological examination and HR-HPV testing and the same indications as above for colposcopy and biopsy. In the present study, 455 women with initial normal smears and without any HR-HPV infection had also randomly a colposcopic examination. There were not particular selection criteria for colposcopy. The primary end point of our study was the detection of a histologically proven HSIL at the biopsy and/or on the LEEP specimen.

### HPV testing

When conventional cytology was performed, specimens for HPV DNA testing were suspended in 1 ml of ViraPap/Viratype transport medium (Digene, Gaithersburg, MD, USA) and stored at −20°C until further processing. When samples were used for liquid-based cytology, 4 ml of the sample were centrifuged and the cell pellet was resuspended in 200 μl of phosphate-buffered saline for HPV testing. Human papillomavirus DNA detection was performed by the commercially available HC-II System (Digene). All scrapes were analysed for the presence of HR-HPV types 16, 18, 31, 33, 35, 39, 45, 51, 52, 56, 58, 59 and 68. This enzyme-linked immunosorbent assay is based on a sandwich hybridisation followed by a nonradioactive alkaline phosphatase reaction with chemoluminescence in microplates. The chosen positive threshold of this test was 1.0 pg ml^−1^ of HPV DNA.

Samples were classified as positive for HR-HPV DNA if the relative light unit (RLU) reading obtained from the luminometer was equal or greater than the mean of the three positive control values supplied by the HC-II kit. As some authors have reported that increasing HPV DNA levels of HR-HPV types were the principal predictors of CIN (Cox *et al*, 1995), we used, as proposed, the ratio RLU/positive controls values to quantify HR-HPV DNA in our samples. Moreover, we added other positive controls such as SiHa cell lines (one to two copies of HPV type 16 per cell) to check the reproducibility of the HC-II sensitivity.

## STATISTICAL METHODS

The statistical methods used were mostly descriptive. Positive predictive value (PPV) and NPV were determined by comparing the results of each test to the gold standard of histology. A few HSIL may have been missed if they were negative on both tests, and thus women were not referred for colposcopy. Using either binomial or normal distribution, 95% percent confidence interval (95% CI) for these values were assessed according to the data. Overall occurrence of HSIL and carcinoma was assessed by Kaplan–Meier analysis with the Mantel Cox's log-rank score for determining statistical significance. Moreover, differences between HR-HPV detection and cytological diagnosis values were compared using Fisher's exact test or *χ*^2^ statistics as adequate, with a *P*-value set to 5%.

## RESULTS

The results are summarised in Table 1^3^

**Table 1:** Follow-up of women with initial normal smears with negative HR-HPV testing (N−)

**Follow-up**	**N−/N−**	**Three or more N−**	**N−/N−/X**	**N−/X**	**HG+**	**Number of women**
12–23 months	750	19	2	54	1	825
24–35 months	1325	125	11	151	2	1612
36–47 months	662	285	42	120	2	1109
48–59 months	124	224	39	39	0	426
60–72 months	92	303	16	18	0	429
	2953 (67.1%)	956 (21.7%)	110 (2.5%)	382 (8.7%)	5	4401

HR-HPV=high-risk human papillomaviruses; HSIL=high-grade squamous intraepithelial lesion. X corresponds to the apparition of cytological abnormalities (from ASCUS to HSIL) and/or a positive HR-HPV testing. HG+ corresponds to the histologically proven four HSIL and one microinvasive carcinoma. These lesions have been detected only in the N−/X cohort.

, 2

**Table 2:** Follow-up of women >29 years with initial normal smears HR-HPV negative (N−)

**Follow-up**	**N−/N−**	**Three or more N−**	**N−/N−/X**	**N−/X**	**HG+**	**Number of women**
12–23 months	557	15	2	38	1	612
24–35 months	1115	94	8	118	2	1335
36–47 months	557	226	30	87	0	900
48–59 months	94	182	27	26	0	329
60–72 months	69	251	15	15	0	350
	2392 (67.8%)	768 (21.8%)	82 (2.3%)	284 (8.0%)	3	3526

HR-HPV=high-risk human papillomaviruses; HSIL=high-grade squamous intraepithelial lesion. X corresponds to the apparition of cytological abnormalities (from ASCUS to HSIL) and/or a positive HPV testing. HG+ corresponds to the histologically proven two HSIL and one microinvasive carcinoma. These lesions have been detected only in the N−/X cohort.

, 3

**Table 3:** Follow-up of women >49 years with initial normal smears HR-HPV negative (N−)

**Follow-up**	**N−/N−**	**Three or more N−**	**N−/N−/X**	**N−/X**	**HG**	**Number of women**
12–23 months	197	4	0	8	0	209
24–35 months	417	32	2	52	0	503
36–47 months	198	87	7	23	0	315
48–59 months	21	49	9	8	0	87
60–72 months	7	48	2	1	0	58
	840 (71.7%)	220 (18.8%)	20 (1.7%)	92 (7.8%)	0	1172

HR-HPV=high-risk human papillomaviruses; HSIL=high-grade squamous intraepithelial lesion. X corresponds to the apparition of cytological abnormalities (from ASCUS to HSIL) and/or a positive HR-HPV testing. HG corresponds to histologically proven HSIL.

. In our series, 4019 women (3242 women >29 years; 1080 women >49 years) had two consecutive normal smears without any HR-HPV infection detected within 12–70 months (median=32 months). In total, 2953 women had only two smears (2392 women >29 years; 840 women >49 years). Of these, 956 women (768 women >29 years; 220 women >49 years) had three or more normal smears without any HR-HPV infection. In all, 110 women (82 women >29 years; 20 women >49 years) after two normal smears without HR-HPV infection presented an HR-HPV infection and/or cytological abnormalities (ASCUS and LSIL). No HSIL was detected in the follow-up of all these women. Moreover, the colposcopic examination of 455 women with persistent normal smears without any HR-HPV infection performed 24–71 months after enrolment (median=37 months) failed to detect histological lesions, even in the eight cases evocative of viral infection by colposcopy.

In total, 382 women (284 women >29 years; 92 women >49 years) developed an HR-HPV infection and/or cytological abnormalities at the second control (median=37 months). At the present time, 136 had only two smears (97 women >29 years; 30 women >49 years). In two women (34 and 35 years old), cytology-detected HSIL and an HR-HPV infection was present at the second control, at 17 and 24 months, respectively. These cytological HSIL corresponded at the histological examination to one HSIL and one microinvasive carcinoma, discovered 17 months after the first normal smear. According to our protocol of follow-up after the discovery of an HR-HPV infection in normal smears, two HR-HPV-positive HSIL were diagnosed at the cytological and histological examinations at a third control at 31 months in a 45-year-old woman and at 38 months in a 25-year-old women. One HR-HPV-positive HSIL was detected at a fourth control by cytology and histology at 41 months in a 26-year-old woman.

In the total follow-up, cytological abnormalities were detected in 140 women (3.2%) of the total population, in 96 women >29 years (2.7%) and in 27 women >49 years (2.3%). HR-HPV infection occurred in the follow-up of 116 women <30 years (13.2%, with 63.3% of regression) and in 323 women >29 years (9.2%, with 70.4% of regression).

Thus, the global NPV for the development of an HSIL is 99.9% (95% CI: 99.8–100%) in the general population, 99.9% (95% CI: 99.8–100%) in women >29 years and 100% in women >49 years. If we consider the follow-up obtained on >5 years of 96 women with two initial consecutive negative HR-HPV testing at 12–24 months of interval, the NPV for the development of an HSIL is 100% whatever the age of the women. In contrast, the PPV of a positive HR-HPV test following an initial normal smear without HR-HPV infection for the detection of an HSIL is 1.1% (95% CI: 0.1–2.1%).

For comparison, if we consider, in our total cohort of 4986 women (3931 women >29 years), attending routine screening with normal smears whatever the HPV testing with a similar protocol of follow-up on a period from 12 to 72 months, 34 HSIL were detected (19 in women >29 years). Thus, for this cohort, the NPV of a normal smear alone for the development of an HSIL is 99.3% (95% CI: 99.2–99.4%) for the total population and 99.5% (95% CI: 99.4–99.6%) for the women >29 years. Moreover, if we consider a similar cohort of 5592 women without limitations of time in the follow-up, we have detected at the present time 71 HSIL (46 HSIL in 4321 women >29 years), which gives an NPV of 98.7% (95% CI: 98.5–98.9%) in the total population and an NPV of 98.9% (95% CI: 98.6–99.2%) in women >29 years. Indeed, 32 HSIL (24 HSIL in women >29 years) have been discovered after an initial positive HPV test before 12 months of follow-up.

**Figure 2:**
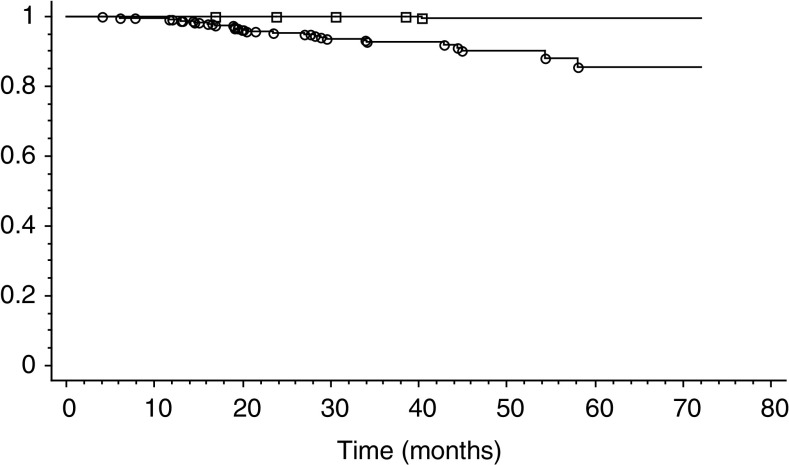
Kaplan–Meier analysis for the occurrence of an HSIL in the whole population. Squares: women with initial negative HR-HPV testing. Circles: women with initial positive HR-HPV testing. *P*<0.001.

**Figure 3:**
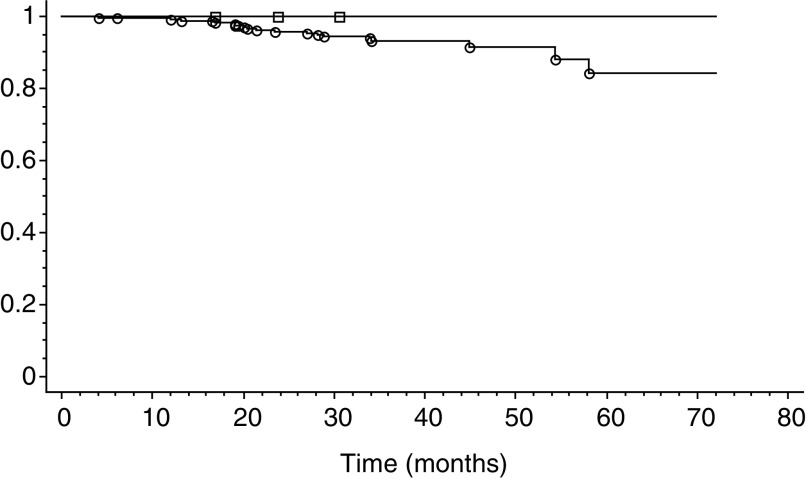
Kaplan–Meier analysis for the occurrence of an HSIL in the population of women >29 years. Squares: women with initial negative HR-HPV testing. Circles: women with initial positive HR-HPV testing. *P*<0.001.

The Kaplan–Meier curves comparing the occurrence of HSIL and carcinoma in the total population (Figure 2

), in women >29 years (Figure 3

), also clearly demonstrate that there is a significant difference (*P*<0.001) between the populations with initial smears with HR-HPV-positive testing compared to populations with a negative test.

## DISCUSSION

This work is at the present time the only longitudinal study using the HC-II assay in association with cervical smears for the follow-up of women with both negative tests at enrolment. Indeed, in the work of Kjaer *et al* (2002) on women <29 years, PCR testing for the detection of HPV was performed on specimens collected into phosphate-buffered saline, and the authors focused their study on women with normal smears and HPV infection. In a related study, the work of Sherman *et al* (2003) was conducted with HC-II assay on cervicovaginal lavage specimens, a technique that is not as good for sampling the endocervical canal as direct brushing. These authors detected 48 HSIL (0.3%) in their 10 years follow-up of 17 594 women with normal smears, but HPV testing results were not used for any aspect of the clinical management or follow-up of the women. Our present study represents a longitudinal experience on 4401 women with different intervals and modalities of follow-up. The protocol that we applied after the discovery of an HR-HPV infection in women with normal smears clearly induces a bias increasing the number of HSIL early detected and largely explains the absence of detection of such lesions after 4 years of follow-up. It is equally true that we cannot also exclude that some cervical lesions, even HSIL, have spontaneously regressed during the mean time of follow-up, especially when two consecutive controls were distant >5 years. Moreover, we obtained long time follow-up >5 years data for 429 women and additional HSIL may be detected with a more extensive study. However, at the present time, our study gives a global NPV of 99.9% for the development of an HSIL and carcinoma in women with an initial smear within normal limits and a negative HR-HPV test result. The occurrence of cytological abnormalities in 3.2% of our population is also lower than the percentage of 5.3% of abnormal smears reported in the literature in general primary screening (Stoler, 2000) (5.2% in our own laboratory). Moreover, if we consider our own previous results on women with normal smears and a positive HR-HPV test with a follow-up, the PPV for detecting an HSIL is 7.7% for one test and 21.2% for recurrent HR-HPV infection (Bory *et al*, 2002). This PPV is only of 1.1% when a previous smear is normal without any HR-HPV infection detected. Thus, the women with double-negative tests really represent a low-risk population for the development of HSIL.

In our series, we detected four HSIL and one microinvasive carcinoma in the follow-up within 17–41 months after the initial normal smear. These HSIL were all CIN3. They were all positive for HR-HPV testing at the time of diagnosis. The initial smears were reviewed. They were all adequate and the first cytological diagnosis was confirmed. The viral load of the initial smear expressed semiquantitatively by the HC-II was low and under the threshold of 1.0 pg ml^−1^ in all cases, and thus the negative HR-HPV test was also confirmed. Despite decades of study, the natural history of cervical intraepithelial lesions is still not completely understood. The short time of evolution for the apparition of these lesions, particularly the microinvasive carcinoma in our series, may correspond to a rapid and direct transformation of the cervical cells by integrated HR-HPV DNA, which may be initially undetectable by the HC-II assay. Indeed, prospective studies suggest that some HSIL can develop independently from low-grade lesions. Koutsky *et al* (1992) found that most cases of HSIL arose *de novo* in the absence of a cytologically detectable low-grade lesion. In the same way, Nobbenhuis *et al* (1999) reported that 88% of the incident cases of cervical intraepithelial lesions were first identified as HSIL. Thus, the few HSIL observed in our series could develop *de novo* at the squamoglandular epithelial junction of the cervix with a particular rapid evolutive profile, and this may explain that both initial cytology and HPV testing have no predictive value in these cases.

In our study, we have not selected women according to their age for the follow-up. The American Cancer Society (Saslow *et al*, 2002) and recently the American College of Obstetricians and Gynaecologists have recommended in their guidelines adjunctive HPV testing in women aged 30 years or older for primary screening. They consider that transient HPV infections are common in women younger than 30 years and a positive test result may lead to unnecessary additional evaluation and treatment. However, in our series, if the prevalence of HR-HPV infections in the follow-up is significantly higher in women <30 years (13.2 *vs* 9.2% in women of 30 years and older, *P*<0.001), at the present time, the percentage of transient infection is identical in these two populations (63.3% in women <30 years *vs* 70.4% in women of 30 years and older). Moreover, we have to emphasise that two out of the five lesions detected in our general population were in 25- and 26-year-old women. Thus, in our experience, the NPV is not different in women >29 years compared to the general population. In another way, if we consider the low prevalence of HSIL in women >50 years attending a regular screening until this age, a double-negative test with an NPV of 100% in this cohort of women would allow a larger interval of screening up to 8 years in this population.

In conclusion, our experience confirms the high NPV of 99.9% for the development of an HSIL in women with normal smears and a negative HR-HPV test with the HC-II assay. If we compare these results with the NPV of a normal smear alone on the same period of follow-up, there is a significant difference in the number of HSIL and carcinoma detected (five *vs* in the double-negative women *vs* 34 for the total population). Our use of HPV testing in primary screening and our protocol of follow-up clearly explains the high number of HSIL discovered in women with initial normal smears in the period between 12 and 72 months. However, if we take into account the additional 32 HSIL (24 HSIL in women >29 years) discovered before 12 months of follow-up on a cohort of 5592 women, according to our protocol, the NPV dramatically decreases to 98.7%. The Kaplan–Meier curves comparing the occurrence of HSIL and carcinoma in women with normal smears positive for HPV testing and women with a negative HPV test clearly confirm these results. Thus, the use of HPV testing combined with cytology significantly reduces the limitations of cytology alone. There is always a limited false-negative fraction represented by the five lesions detected in our series. This fraction could be reduced and eventually abolished if we consider a second negative HR-HPV test 1–2 years after the initial test, which gives an NPV of 100% in our experience, but this policy will generate a higher cost. In any case, all these women could be safely screened at longer intervals between 3 and 5 years. This policy will offset the increased costs induced by an additional HR-HPV testing in primary screening.
